# Development of a rapid loop-mediated isothermal amplification assay for diagnosis and assessment of cure of *Leishmania* infection

**DOI:** 10.1186/s12879-017-2318-8

**Published:** 2017-03-23

**Authors:** Sandeep Verma, Ruchi Singh, Vanila Sharma, Ram Avtar Bumb, Narendra Singh Negi, V Ramesh, Poonam Salotra

**Affiliations:** 1 National Institute of Pathology (ICMR), Safdarjung Hospital Campus, New Delhi, 110029 India; 2 Department of Skin, STD and Leprosy, S. P. Medical College, Bikaner, India; 3 Department of Medicine, Safdarjung Hospital, New Delhi, India; 4 Department of Dermatology, Safdarjung Hospital, New Delhi, India

**Keywords:** *Leishmania*, VL, PKDL, Diagnosis, LAMP

## Abstract

**Background:**

Leishmaniasis is a spectrum of diseases with great relevance to public health. Conventional diagnostic methods are time consuming, needing trained personnel. A robust, rapid and cost effective diagnostic test is warranted for on-time diagnosis and field application.

**Methods:**

We have developed a loop mediated isothermal amplification (LAMP) assay with primers (*n* = 6) based on *Leishmania donovani* kDNA for detection of *Leishmania* infection, using a closed tube to prevent cross-contamination. The assay was used to detect Leishmania infection in biological samples obtained from patients of visceral leishmaniasis (VL), post kala-azar dermal leishmaniasis (PKDL) and cutaneous leishmaniasis (CL).

**Results:**

The assay was positive for *L. donovani*, *L. tropica* and *L. major* parasites, with the highest sensitivity towards *L. donovani* (1 fg DNA). The high sensitivity of the assay for detection of *L. donovani* was reflected in its ability to detect parasite DNA within 30 min of amplification time with a threshold detection limit of ≥25 copies per reaction. The assay detected parasite in 64 of 66 VL blood samples (sensitivity, 96.9%; 95% CI: 89.6-99.2%), 15 of 15 VL bone marrow aspirate samples (sensitivity, 100%; 95% CI:79.6-100%), 65 of 67 PKDL tissue biopsy samples (sensitivity, 97%; 95% CI:89.7-99.2%). The assay was evaluated in a few cases of CL wherein it was found positive in 8 of 10 tissue biopsies (sensitivity, 80%; 95% CI: 49-94.3%). The assay was negative in all control blood (*n* = 76) and tissue biopsy (*n* = 24) samples (specificity, 100%; 95% CI: 96.3-100%). Further, the assay was evaluated for its utility in assessment of cure in treated VL and PKDL patients. The assay detected parasite DNA in 2 of 20VL blood samples and 2 of 21 PKDL tissue samples. Out of 4 cases that were positive for parasite DNA at post treatment stage, 2 patients (1VL and 1 PKDL) returned with relapse.

**Conclusions:**

The study demonstrated a *Leishmania* genus specific closed tube LAMP assay for reliable and rapid molecular diagnosis of VL and PKDL with potential for application in assessment of cure.

## Background

Leishmaniasis, a group of diseases caused by protozoan parasite *Leishmania* is transmitted to humans by the bite of infected phlebotomine female sandflies. The disease is endemic in about 98 countries, affecting 12 million people worldwide. Clinical manifestations include cutaneous leishmaniasis (CL), mucocutaneous leishmaniasis (MCL) to the deadly visceral leishmaniasis (VL). VL mostly prevalent in tropical regions is caused by the parasites of *L. donovani* complex. In 2014, more than 90% of new cases of VL reported to WHO occurred in six countries: Brazil, Ethiopia, India, Somalia, South Sudan and Sudan [1]. Post kala-azar dermal leishmaniasis (PKDL) is a neglected complication of VL, characterized by macular, maculopapular and nodular lesions in a patient who has recovered from VL. In India and Bangladesh, PKDL is reported in 5-15% of patients treated for VL, usually after an interval of a few months to several years [2, 3], however in Sudan 50-60% of VL cases develop PKDL within a few weeks of treatment [4]. The need to identify persons affected with PKDL and treat them as a part of VL control programs have been emphasized since PKDL patients are considered as an important reservoir for the parasite during inter-epidemic periods of VL [4]. In India, CL is primarily endemic in the western Thar region of Rajasthan, particularly in Bikaner, where *L. tropica* is the major causative agent [5].

Microscopic examination of tissue smears is considered as the gold standard for the definitive diagnosis of VL and PKDL that suffers from low sensitivity. The sensitivity of microscopy for VL ranges from 93-99% for spleen aspirate to 53-86% for bone marrow, and 53-65% for lymph node aspirates [6] while for PKDL diagnosis it is higher for nodular cases (67-100%) compared to papular (36-69%) or macular (7-33%) cases [7]. Serological methods (rK39 strip test, enzyme-linked immunosorbent assay, direct agglutination test etc.) are highly sensitive, however, fail to distinguish between past and present infections hence are not conclusive for PKDL diagnosis [8–11]. In last several years advances in the polymerase chain reaction (PCR) based methods have been introduced for diagnosis of leishmaniasis [12–15]. Several PCR protocols have been developed for detection of *Leishmania* parasite, such as triplex PCR [16], multiplex PCR [17], restriction fragment length polymorphism (RFLP) analysis and nested PCR [18]. Quantitative Real-time PCR (Q-PCR) has emerged as a highly sensitive tool for VL and PKDL diagnosis with simultaneous quantification of the parasite burden [19–22]. However, these assays are not used for routine diagnosis of *Leishmania* infection as these require of well equipped laboratory facilities along with technical expertise.

Loop-mediated isothermal amplification (LAMP) assay, a method to amplify DNA with rapidity and high specificity under isothermal conditions, has emerged as an efficient tool in the field of diagnostics [23]. LAMP assay is rapid and cost effective as compared to PCR, offering improved sensitivity and a higher tolerance for inhibitors present in a number of clinical samples [24, 25]. Shorter reaction time with visual judgment of positivity without requiring sophisticated equipments makes it an attractive diagnostic method for field application.

Although, a few studies employing LAMP assay to diagnose *Leishmania* infection are known [26–32] however, these assays are limited in their utility because of the false positivity due to cross contamination [29], or prolonged reaction time [31] or the use specialized equipment [32]. Here, we have developed a LAMP assay based on kinetoplast minicircle DNA (kDNA) of *L.donovani* for reliable and rapid diagnosis of *Leishmania* infection in a closed tube manner using SYBR Green I for detection of the amplified product.

## Methods

### Parasite DNA

DNA from different *Leishmania* spp. including *L. donovani* AG83 (MHOM/IN/83/AG83), *L. major* ASKH (MHOM/SU/73/5ASKH) and *L. tropica* WR 683 (MHOM/ SU/58/OD) were used in the study. All parasite cultures were propagated in medium 199 supplemented with 25 mM HEPES, pH 7.5 and 10% fetal calf serum. Parasites were harvested in the late log phase and washed in phosphate-buffered saline prior to DNA isolation using Promega genomic wizard DNA isolation kit (Promega Corporation, USA) according to the manufacturer’s instructions.

### Clinical samples

VL and PKDL patients originating from Bihar and reporting respectively to Departments of Medicine and Dermatology, Safdarjung Hospital, New Delhi, were included in this study. The patients with characteristic symptoms of VL (fever, hepatosplenomegaly, anemia and leucopenia) and PKDL (on clinico-histopathological observations) who were positive by rK39 strip test were included in this study. All the cases were confirmed by Q-PCR assay [21]. Peripheral blood (0.5 ml from 66 cases) and bone marrow aspirate BMA (100 μl from 15 cases) were collected from VL patients while tissue biopsy (4-6 mm punch biopsy from 67 cases) were collected from PKDL patients at pre-treatment stage. VL and PKDL samples used in this study included those reported earlier [29] in addition to new cases that reported thereafter. Tissue biopsy sample DNA of Q-PCR [5] confirmed CL (*n* = 10) cases who reported to Department of Skin, STD &Leprosy, S.P. Medical College, Bikaner (Rajasthan), India, were also included in this study. Blood (0.5 ml) samples from healthy volunteers (endemic *n* = 24; non-endemic, *n* = 38), malaria patients (*n* = 7) and tuberculosis patients (*n* = 7) as well as tissue biopsy (4-6 mm punch biopsy) samples from leprosy patients (*n* = 18) and cases of fungal diseases (Sporotrichosis or *Pityriasis lichenoides chronica*) (*n* = 6) were included as controls.

### DNA isolation from clinical samples

Blood and bone marrow sample was collected in heparinized tubes. DNA extraction was performed using QIAamp DNA Blood mini kit (Qiagen, Germany) according to manufacturer’s instructions. DNA was isolated from 200 μL of blood and eluted in 50 μL nuclease free water. In case of BMA sample, 100 μL of BMA was used and eluted in 100 μL nuclease free water. In order to achieve maximum yield in BMA samples, digestion was performed overnight with proteinase K in lysis solution [29]. Punch biopsy (4-6 mm) sample was taken under sterile conditions from the dermal lesions of PKDL and CL cases in NET buffer (150 mM NaCl, 15 mM Tris-HCl pH 8.30 and 1 mM EDTA). DNA was extracted and eluted in 100 μl of nuclease free water using QIAamp DNA Tissue kit according to manufacturer’s instructions.

### Design of LAMP primers

**Fig 1:**
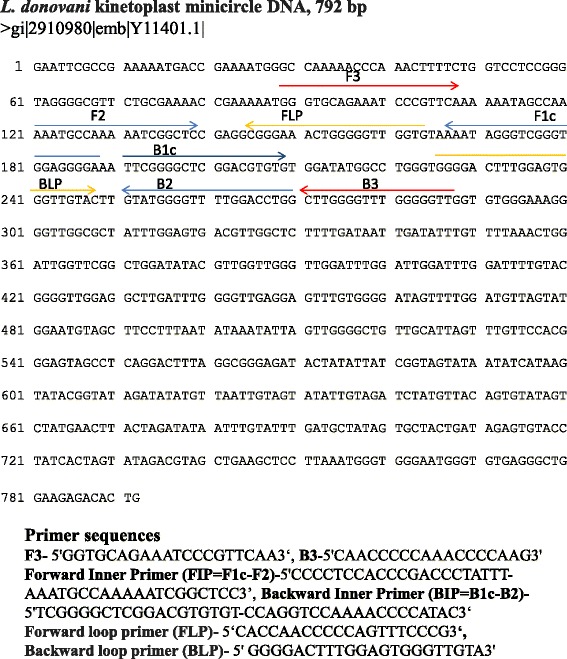
Primers for LAMP assay. Primers for LAMP assay were designed for amplification of *Leishmania* DNA from *L. donovani* kinetoplast minicircle sequence (Acession no Y11401) using Primer Explorer V4 software. A total of six primers (F3, B3, FIP, BIP, FLP and BLP) were designed for the LAMP assay

Sets of outer forward primer (F3), outer backward primer (B3), forward inner primer (FIP) and backward inner primer (BIP) targeting regions of *L. donovani* kDNA sequence (GenBank accession no. Y11401.1) were designed using the online Primer Explorer V4 software (Eiken Chemical Co. Ltd., Japan, http://primerexplorer.jp/elamp4.0.0/index.html). Loop primers [Forward loop primer (FLP) and Backward loop primer (BLP)] were designed manually. Three primer sets were tested and the primer set giving highest sensitivity was used in the study. The primer set used is shown in Fig. 1.

### Cloning and sequencing of LAMP amplicon from *Leishmania spp*

The LAMP amplicon from kDNA minicircle from *Leishmania spp*, was amplified using F3 and B3 primers. The PCR reaction mixture of final volume 50 μl contained 50 pmol of F3 and B3 primers, 10 ng of parasite DNA (*L. donovani, L. major* and *L. tropica*), 200 *μ*M each of dNTP mix, 2 mM MgSO4, and 1 U Platinum Taq DNA polymerase (Invitrogen, USA) in 1× Taq buffer. PCR reaction conditions included initial denaturation at 94 °C for 2 min., followed by 35 cycles of 94 °C for 15 s., 62 °C for 30 s., 68 °C for 30 s., and final extension at 68 °C for 2 min. The PCR amplicon (200 bp) along with 100 bp DNA ladder were resolved by 2% agarose gel electrophoresis. PCR amplicons were purified and ligated into pGEMT easy cloning vector (Promega Corporation, USA) following manufacturer's instructions. Recombinant plasmids were characterized using restriction enzyme EcoRI (MBI Fermentas, USA) for insert fall out (200 bp). The amplicons were cloned into pGEMT vector and transformed into *E.coli* DH5α strains. The positive clones were screened by blue and white selection on X-gal and IPTG containing Luria-Bertani agar plates. The white colonies were selected and sequenced using T7/SP6 primers on Automated Sequencer 3730 Version 3.0 (ABI PRISM).

### LAMP assay

The LAMP reaction was performed in 25 μl of reaction mixture containing 40 pmol each of FIP and BIP primers, 5 pmol each of F3 and B3 primers,20 pmol each of the FLP and BLP, 1.4 mM of each deoxynucleoside triphosphate, 0.8 M betaine, 20 mM Tris-HCl, pH 8.8, 10 mMKCl, 10 mM (NH_4_)_2_SO_4_, 8 mM MgSO_4_, 0.1% TritonX-100, 8 units of *Bst* DNA polymerase large Fragment (New England Biolabs, Ipswich, MA), and 2 μl of DNA from parasites and clinical specimen. Before starting the reaction, 1 μl of diluted (1/10) SYBR Green I (Molecular Probes, Eugene, OR, USA) was placed on the inner side of the cap of PCR tube. The tube was closed and incubated at 65 °C for 30 min in a heating block. On completion of reaction, the tube was centrifuged to allow mixing of SYBR Green I with the amplified product. All positive samples produced a green colour almost immediately upon mixing of SYBR Green I, while the negatives remained orange. The samples were tested in a batch of 24 including clinical samples from patients of Leishmaniasis and other diseases. In every batch, minimum 2 DNA samples from healthy individuals and one blank control in which DNA was replaced by water was used as negative and reagent control, respectively.

### Quantitative Real Time PCR (Q-PCR) assay

SYBR Green I based *Leishmania* genus specific Q-PCR was performed using F3 and B3 primers using the reaction conditions as described previously [21]. A 10 μl of the PCR reaction was performed, consisting of 1X SYBR Green I PCR Master mix (Applied Biosystems, USA), 5 pmol forward primer F3, 5 pmol reverse primer B3, and 1 μl sample DNA. The cycling parameters were 50 °C for 2 min, 95 °C for 10 min, and 40 cycles of 95 °C for 15 s and 60 °C for 1 min. A standard curve was constructed using 10-fold serially diluted kDNA plasmid corresponding to 10^8^ to 10^3^ copies per reaction. A threshold cycle value (Ct) was calculated for each sample by determining the point at which the fluorescence exceeded the threshold limit. All the reactions were performed in triplicate.

## Results

### Cloning and sequence analysis of kDNA minicircle

**Fig 2:**
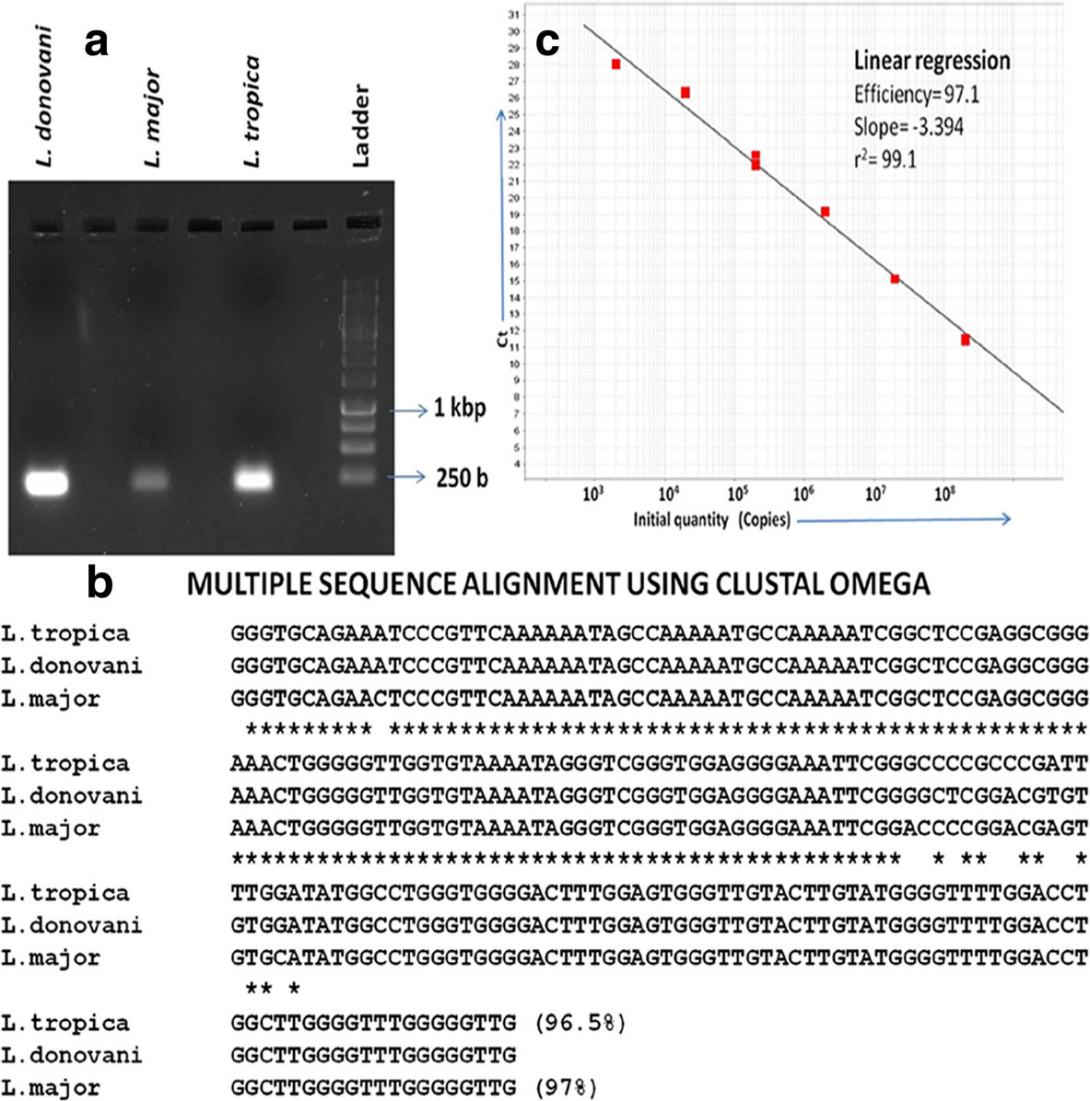
PCR amplification, multiple sequence alignment and standard curve plot. **a** PCR amplification of kDNA minicircle using parasite DNA from *L. donovani, L. major* and *L.tropica*. Product of 200 bp size was obtained in all cases. **b** Multiple Sequence Alignment using CLUSTAL OMEGA. The amplified kDNA sequences from *L. donovani*, *L. tropica* and *L. major* were sequenced and percentage identity analyzed using Clustal Omega (http://www.ebi.ac.uk/Tools/msa/clustalo/). Percent identity is shown in parenthesis. **c** Standard curve obtained with serially diluted *L. donovani* kDNA plasmid. SYBR Green 1 based QPCR was carried out using F3 and B3 primers at each dilution. Graph shows Ct value plotted at different copy number of kDNA

PCR-amplification of kDNA from total DNA isolated from *L. donovani*, *L. major* and *L. tropica*, using F3 and B3 primers yielded the product of 200 bp size of variable intensity (Fig. 2a). The corresponding amplicons were cloned, sequenced and subjected to multiple sequence alignment. Data revealed 100% sequence identity in case of *L. donovani,* 97% in case of *L. major* and 96.5% in case of *L. tropica* (Fig. 2b).

### Determination of kDNA copy number in *Leishmania spp*. using Q-PCR

For evaluation of differences in copy number of kDNA minicircle in different *Leishmania* spp, a standard curve was established with serially diluted known number of kDNA plasmid copies (10^3^ to 10^8^) from *L. donovani*. The mean standard curve was linear over 6 log range with correlation coefficient (r^2^) of 0.991 (Fig. 2c). A negative control (water instead of template DNA) with each PCR assay was included for stringent measures. Q-PCR assay with parasite DNA from *Leishmania spp.* revealed that the copy number of kDNA varied from 500-10000 copies in *L. donovani,* 10-20 copies in *L. tropica* and 1-10 copies in *L. major*.

### Analytical sensitivity and specificity of the LAMP assay with *Leishmania spp*. DNA

**Fig 3:**
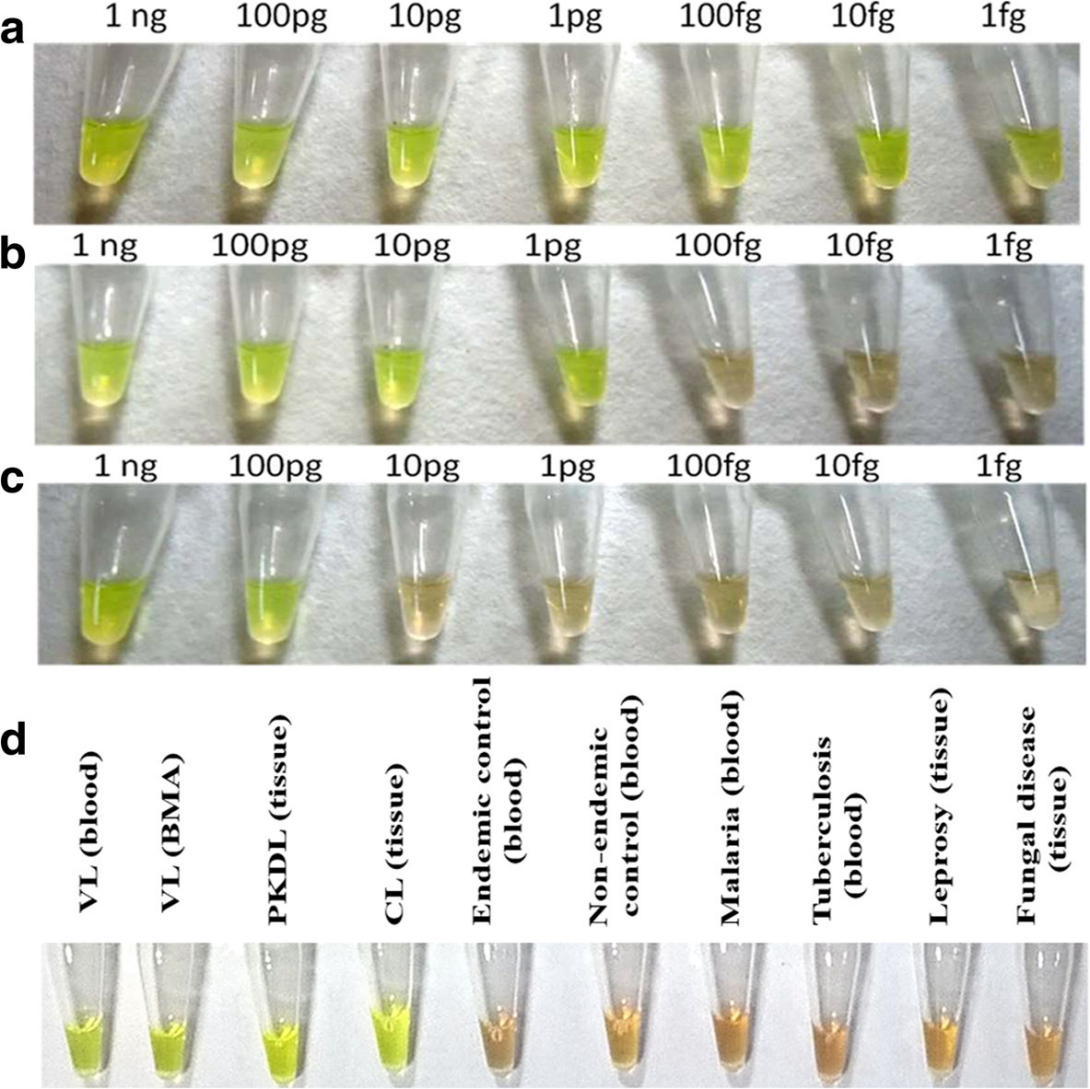
Sensitivity and specificity of LAMP assay. Sensitivity of LAMP assay using serial dilutions of DNA (1 ng–1 fg) from **a** *L. donovani* **b** *L. tropica* and **c** *L. major*. **d** Specificity of LAMP assay for diagnosis of VL, PKDL and CL in clinical samples

The assay was performed with DNA from *L. donovani*, *L. tropica* and *L. major* parasites and found positive in all. Specificity of the assay was evaluated using DNA from microorganisms causative of the common infectious diseases prevalent in India such as *Plasmodium spp.*, *Mycobacterium leprae* and *M. tuberculosis* which were all observed to be negative. Further, the analytical sensitivity of the LAMP assay was determined by serial dilution of parasite DNA (10 ng – 1 fg) from different *Leishmania spp*. The sensitivity of LAMP assay was higher towards *L. donovani* (up to 1 fg parasite DNA) and *L. tropica* (up to 1 pg parasite DNA) as compared to *L. major* (up to 100 pg parasite DNA), (Fig. 3a-c). These results correlated well with the intensity of PCR product obtained with *L. donovani*, *L. tropica* and *L. major* (Fig. 2a). The sensitivity of the assay was also determined using DNA isolated from *L. donovani* parasites (10^5^ to 10) spiked healthy blood (200 μl) and,the LAMP assay could detect the *Leishmania* DNA up to 10 parasites dilution.

### Comparison of detection limits of LAMP and Q-PCR assays

To assess the threshold detection limit for LAMP and Q-PCR assays for *L. donovani,* the two assays were performed with serial dilutions of kDNA plasmid. The LAMP assay was found positive for ≥25 copies per reaction whereas Q-PCR assay was positive for ≥10 copies per reaction.

### Sensitivity and specificity of the LAMP assay in clinical samples

The clinical applicability of the LAMP assay was assessed using DNA isolated from the clinical samples including blood from cases of VL tuberculosis, malaria and tissue biopsy from cases of PKDL, CL and leprosy. The assay was positive for VL, PKDL and CL samples and negative for all others (Fig. 3d). The LAMP assay was positive in 64 out of 66 confirmed cases of VL using DNA from blood samples, giving a sensitivity of 96.9% (95% CI, 89.6-99.2%). In comparison, the microscopy was positive in 42 of 66 (63.6%) cases. The assay was positive in all (*n* = 15) BMA samples of confirmed VL cases at pre-treatment stage giving sensitivity of 100% (95% CI, 79.6-100%). LAMP assay detected parasite DNA in 65 out of 67 confirmed PKDL cases using tissue biopsy, giving sensitivity of 97% (95% CI, 89.7-99.2%) while microscopy was positive in only 21 out of 67 (31.3%) cases. When evaluated in CL cases, the assay was found positive in 8 of 10 cases giving sensitivity of 80% (95% CI: 49-94.3%). The assay was negative in all (*n* = 100) controls (blood, *n* = 76; tissue, *n* = 24) giving a specificity of 100% (95% CI, 96.3 -100%). These results revealed a high sensitivity (Table 1) and specificity (Fig. 3d) of LAMP assay for diagnosis of VL and PKDL.

**Table 1:** Sensitivity of the LAMP assay for diagnosis of VL, PKDL and CL

Sample	Cases tested for LAMP (confirmed by Q-PCR^ab^)	Cases positive for LAMP	LAMP Sensitivity (95% Confidence Interval)
VL (Blood)	66^a^	64	96.9% (89.6 - 99.2%)
VL (BMA)	15^a^	15	100% (79.6 - 100%)
PKDL (Tissue)	67^a^	65	97% (89.7 - 99.2%)
CL (Tissue)	10^b^	8	80% (49 - 94.3%)

^a^Reference no. 20

^b^Reference no. 21

### Assessment of novel LAMP assay as test of cure

To evaluate the applicability of LAMP assay for assessment of cure in VL and PKDL, 20 cases of VL and 21 cases of PKDL were examined 1-month post-treatment, as reported in our earlier study employing Q-PCR (21, 33). When examined at post treatment stage, the VL blood (2 of 20) samples and PKDL tissue (2 of 21) samples from the follow-up samples of the above patients were found to be positive for parasite DNA both by LAMP and QPCR assay. This confirmed that the LAMP and Q-PCR assay correlated well for detection of parasite in clinical samples. Out of the four cases (2 VL and 2 PKDL) that were positive for parasite at post-treatment stage, two patients (1 VL and 1 PKDL) appeared with relapse.

## Discussion

An effective control of VL and PKDL warrants reliable, rapid and cost effective diagnostic tools. Diagnosis by microscopy is a classical confirmatory test that suffers from low sensitivity. PCR based diagnosis has remained a definitive breakthrough, however, it is a time consuming process and needs sophisticated instruments. A diagnostic test to be prospected as a point-of-care test and to be used in the field settings must qualify two basic criteria: a) high sensitivity and specificity & b) economical and easy to perform. The widely prevalent rK39 strip test as a diagnostic test is adequate for VL but is of limited value for PKDL diagnosis as a positive result produced may occur due to antibodies persisting due to past episode of VL. Identification of PKDL patients is important to VL Elimination Program [33] as they serve as durable reservoirs during inter-epidemic episodes, hence, a faster and reliable molecular assay for accurate PKDL diagnosis is warranted. Among molecular diagnostic methods LAMP has emerged as a promising test and has been successfully applied in diagnosis of various protozoan diseases like African trypanosomiasis [34], malaria [35], giardiasis [36], cryptosporidiosis [37] and leishmaniasis [26–32]. The closed tube LAMP assay reported here has the potential as a reliable point of care test for diagnosis of PKDL, with minimal risk of cross-contamination. The LAMP assay using 4 primers that we reported earlier for rapid diagnosis of VL and PKDL [29], was species- specific for *L.donovani*, being negative for *L.infantum*, *L. tropica*, and *L. major*. The novel 6 primer LAMP assay reported here is superior to the previous one, being faster and more sensitive for detection of *L. donovani*. Further, it is applicable for diagnosis of CL since it detects *L. tropica* and *L. major*. The assay had the highest sensitivity towards *L. donovani*, being capable to detect 1 fg parasite DNA per reaction that corresponded to less than 1 parasite. The sensitivity and specificity of our LAMP assay is high with a short amplification time. Evaluation of the test in small number of cases of CL gave a sensitivity of 80%; however, a study is warranted in large number of cases to explore its utility for diagnosis of CL.

We sought to address whether the lower sensitivity of detection in *L. major* and *L. tropica* was due to difference in sequence or copy number of the target kDNA. It is well known that all copies of kDNA within a parasite kinetoplast are not identical and variations in the target minicircle sequences are observed. Here, a base difference in the sequence of the chosen minicircle amplicon at the forward primer site in case of *L. major* DNA was observed. This may explain the reduced sensitivity of the assay to detect *L. major* spp. Q-PCR assay showed variations in minicircle copies from 500-10000 in *L. donovani* strains, 10-20 copies in *L. tropica* and 1-10 copies in *L. major*, although, estimates of the copy number for *L. major* is not conclusive due to variation in *L. major* targeted sequence at forward Q-PCR primer site.

In the present study we have chosen Q-PCR (21) as the reference test as it is reported as one of the most sensitive molecular assay to detect parasite DNA in clinical samples, therefore, we compared the threshold detection limit of kDNA copies by LAMP vs. Q-PCR assay. The threshold copy number detection limits for LAMP and Q-PCR assays were determined as ≥25 copies and ≥10 copies, respectively. The comparative detection limits for the two assays were similar to that reported for detection of orf virus [38]. Thus, with the sensitivity comparable to Q-PCR (both detects up to 1 fg DNA), LAMP assay can undoubtedly be volunteered as an efficient tool for diagnosis of VL and PKDL as it is simpler, faster and easy to perform. In addition, LAMP assay efficiently detected parasite DNA in the same VL (*n* = 2/20) and PKDL (*n* = 2/21) cases at post-treatment stage which were detected positive for parasite DNA by Q-PCR as reported earlier [21, 39]. Close agreement in the results of LAMP and Q-PCR assays at post-treatment stage highlights the utility of LAMP assay as a point-of-care test for assessment of cure of VL and PKDL cases, though a detailed evaluation of the assay in a large number the patients is required to appraise its prognostic efficacy. The less invasive methods of sample collection like finger prick blood sampling and direct LAMP assays avoiding the DNA isolation steps are desirable for field based diagnosis.

Despite the numerous advantages, LAMP assay faces a major issue of product cross-contamination that limits its application in the field. In the past, LAMP assays using wax dye capsule method, calcein or hydroxyl-napthol blue metal indicators at the start of the reaction were introduced to prevent opening of lid after amplification [40]. Our closed tube LAMP assay using SYBR green I is a further improvement as this indicator gives better sensitivity and clarity in visualization of the results.

## Conclusions﻿

The LAMP assay developed in the present study can detect DNA from *L. donovani*, *L. tropica* and *L. major* parasites; hence it is applicable for the rapid and reliable diagnosis of VL, PKDL and CL. The method is easy to perform, cost-effective and rapid with much reduced reaction time of 30 mins. Above all, the need to open the tube for adding SYBR Green I for detection of amplification is not required, ruling out the occurrence of false positive results. All the above factors justify the use of the presently defined LAMP assay in large epidemiologic studies to be performed in field settings. To conclude, the LAMP assay developed by us falls in the category of high performance result oriented tests having rapid turn-around time with a minimal chance of error. Thus, we propose that the reformed LAMP assay reported here may be exploited as a point of care test for diagnosis as well as for assessment of cure for both VL and PKDL.
